# Characterizing the trophic niches of stocked and resident cyprinid fishes: consistency in partitioning over time, space and body sizes

**DOI:** 10.1002/ece3.2272

**Published:** 2016-06-26

**Authors:** Tea Bašić, J. Robert Britton

**Affiliations:** ^1^ Centre for Conservation Ecology and Environmental Sciences Faculty of Science and Technology Bournemouth University Poole Dorset BH12 5BB U.K

**Keywords:** *Barbus barbus*, lowland rivers, stable isotope analysis, trophic niche

## Abstract

Hatchery‐reared fish are commonly stocked into freshwaters to enhance recreational angling. As these fishes are often of high trophic position and attain relatively large sizes, they potentially interact with functionally similar resident fishes and modify food‐web structure. Hatchery‐reared barbel *Barbus barbus* are frequently stocked to enhance riverine cyprinid fish communities in Europe; these fish can survive for over 20 years and exceed 8 kg. Here, their trophic consequences for resident fish communities were tested using cohabitation studies, mainly involving chub *Squalius cephalus*, a similarly large‐bodied, omnivorous and long‐lived species. These studies were completed over three spatial scales: pond mesocosms, two streams and three lowland rivers, and used stable isotope analysis. Experiments in mesocosms over 100 days revealed rapid formation of dietary specializations and discrete trophic niches in juvenile *B. barbus* and *S. cephalus*. This niche partitioning between the species was also apparent in the streams over 2 years. In the lowland rivers, where fish were mature individuals within established populations, this pattern was also generally apparent in fishes of much larger body sizes. Thus, the stocking of these hatchery‐reared fish only incurred minor consequences for the trophic ecology of resident fish, with strong patterns of trophic niche partitioning and diet specialization. Application of these results to decision‐making frameworks should enable managers to make objective decisions on whether cyprinid fish should be stocked into lowland rivers according to ecological risk.

## Introduction

The release (stocking) of hatchery‐reared fish into freshwater fisheries remains a widespread management technique used around the world to enhance recreational angling (Cowx 1994; Hunt et al. 2014). It can involve the supplementary stocking of extant species as well as the introduction of nonindigenous species (Antognazza et al. 2016). It is often completed in preference to alternative options to enhance fish communities, such as habitat management (Arlinghaus and Mehner 2005). Given their attraction to anglers through their sporting qualities, stocked fish are often species that grow to relatively large sizes and have high trophic positions (Holmlund and Hammer 2004; Fujitani et al. 2016), such as apex predators (Eby et al. 2006). Correspondingly, stocked fishes can influence the natural functioning of ecosystems through, for example, increasing species richness at higher trophic levels and altering food‐web linkages and complexity (Eby et al. 2006).

Releases of fish into an ecosystem where the resources are not fully exploited can lead to their exploitation of vacant dietary niches that facilitates their integration into the community by minimizing competition with resident fishes (Shea and Chesson 2002; Jackson and Britton 2014; Tran et al. 2015). However, as stocking exercises often involve the enhancement of population sizes of existing species to increase angler catch rates (Cowx 1994), it could lead to increased intra‐ and intercompetition for food resources (Vehanen et al. 2009). The niche variation hypothesis then predicts populations will become more specialized in their diet (Van Valen 1965), resulting in reductions in trophic niche sizes following stocking (Human and Gordon 1996; Olsson et al. 2009). Conversely, increased competition for resources can also result in enlarged population trophic niches that enable species and individuals to maintain their energy requirements by switching to more general diets (Svanbäck and Bolnick 2007). These theoretical perspectives can be used as the basis for testing how stocking can impact the trophic ecology of resident species (Tran et al. 2015).

In European rivers, *B. barbus* are stocked regularly in areas covering both their indigenous and nonindigenous ranges (Antognazza et al. 2016). In England, riverine populations are regularly enhanced with hatchery‐reared fish of between 10 and 25 cm (age 1+ and 2+ years). These either supplement indigenous populations or provide new catch‐and‐release angling opportunities in the nonindigenous range (Wheeler and Jordan 1990). Should these fish survive the stocking process (Bolland et al. 2008, 2009), then they can persist for at least 20 years (Britton et al. 2013), providing considerable benefits to catch‐and‐release recreational angling (Britton and Pegg 2011). While there is some knowledge on the genetic outcomes of *B. barbus* stocking (Antognazza et al. 2016), there is little knowledge on their ecological impacts, This is despite their omnivory, potential for long life spans and individuals attaining weights in excess of 8 kg (Britton and Pegg 2011; Britton et al. 2013). It is also in contrast to knowledge on the impacts of stocked species of the Salmonidae family, where there is substantial information on their impacts on wild stocks (e.g., Ruzzante et al. 2004; Larsen et al. 2015). These impacts include trophic cascades that result from the increased abundance of species in higher trophic positions in the food web (Eby et al. 2006). Unlike cyprinid fish, many stocked salmonids are captured and removed by anglers soon after their stocking, limiting long‐term impacts due to short residence times (Baer et al. 2007). Where these salmonids do survive in the wild, their relatively short life spans can limit their persistence, although ecological and genetic consequences can still accrue (Simon and Townsend 2003; Le Cam et al. 2015).

The aim of this study was to thus quantify the ecological consequences of *B. barbus* stocking for resident fishes through determining their trophic interactions and consequences for somatic growth rates. This was completed over three spatial and timescales, and for fish of a range of body sizes. As *B. barbus* can attain large body sizes and their functional traits favor feeding on the benthos, assessments mainly used cohabitation experiments and field studies involving chub *Squalius cephalus*. This is a similarly large‐bodied, omnivorous and long‐lived species (e.g., Mann 1976) that occurs in sympatry with *B. barbus* in lowland rivers in England. Due to the ecological theory outlined, particularly the niche variation hypothesis (Van Valen 1965), it was predicted that following a stocking event, *B. barbus* and *S. cephalus* will have reduced trophic niche sizes as a result of increased diet specializations, with concomitant decreases in the somatic growth rates of both fishes.

## Materials and Methods

### Pond mesocosms

The pond mesocosm experiment tested the outcomes for the trophic niches and somatic growth rates of both fishes between their allopatric and sympatric contexts. Three treatments were used: both species in allopatry (*n* = 10), and a final treatment where they were present in sympatry (*n* = 5 + 5), with three replicates per treatment. This enabled testing of their trophic niche size and position in allopatry and thus how being in sympatry affected these trophic metrics. All fish used were juveniles, of starting lengths between 60 and 88 mm and sourced from aquaculture.

Each mesocosm comprised of an independent enclosure situated within one larger natural pond (30 × 12 m; 1 m depth). The rationale of the use of enclosures was that they provided uniform habitats across the treatments and replicates in which the fish would be exposed to same prey fauna. As these preys were all located within the larger pond, then their stable isotope values would be similar. Thus, any differences in the stable isotope data of the fishes would be the result of their dietary interactions within the treatments, not due to inherent variability in the stable isotope values of their prey. The enclosures comprised of aluminium frames of 1.66 m (length) × 1.05 m (width) × 1.2 m (height) that were enclosed within a net of 7 mm square mesh that prevented fish movements in and out of the enclosure, but allowed the movement of water and invertebrates. The enclosures were located randomly across the larger pond, with spacing of at least 0.5 m between them to ensure they provided enclosed and independent habitats for each replicate and that were identical at the commencement of the experiment. Antipredator netting (15 mm mesh) was placed over the top of all enclosures. The enclosures were sufficiently heavy that their remained stationary throughout the experimental period without moving and without needing to be tied down. The height of the enclosures meant they settled on the substrate, with macrophytes able to grow within each of them (mainly *Elodea* spp.)

The experiment commenced in May 2014 and ran for 100 days, providing sufficient time for fish dorsal muscle to reach isotopic equilibrium (Jackson et al. 2013; Busst and Britton 2016). The mean water temperature during the experiment was 18.2 ± 0.3°C, measured using a temperature logger in the center of the pond that recorded temperature hourly (TinyTag TGP‐4017). The enclosures were placed into the pond 7 days prior to the start of the experiment and all fish were measured prior to their release (fork length, nearest mm). On day 100, each enclosure was removed from the pond with the fish removed, euthanized (anesthetic overdose, MS‐222) and placed on ice. At the same time, samples of macro‐invertebrates were taken from each enclosure via sorting through the remaining pond substrate and macrophytes. These were mainly Chironomid larvae, but also included *Gammarus pulex*,* Asellus aquaticus* and corixids.

In the laboratory, the fish were remeasured and a sample of dorsal muscle was taken for stable isotope analysis. Their growth rates were calculated as incremental length (IL), determined from (*L*_*t* + 1_ – *L*_*t*_)/*t*, where *L*_*t*_ = initial starting lengths, *L*_*t* + 1_ = total end lengths and *t *= number of days. The macro‐invertebrate samples were sorted to species, enabling three samples per species to be prepared for stable isotope analysis. There was no requirement to sort the species by size, as they were similar in body sizes. Each of these samples comprised of between three and six individuals. A random selection of fish dorsal muscle samples (*n* = 15–18 per species and treatment; minimum number of samples per replicate = 4) was then also selected for stable isotope analysis. All of these samples were then dried at 60°C for 24 h, ground and weighed and analyzed at the Cornell Isotope Laboratory, New York, USA for their stable isotopes of *δ* ^13^C and *δ* ^15^N that were expressed as isotope ratios per mille (‰). For initial analyses, *δ* ^15^N data were transformed to trophic position (TP), using the equation TPi = [(*δ* ^15^N_i_ – *δ* ^15^N_base_)/3.4] + 2, where TP_i_ is the trophic position of the individual fish, *δ* ^15^N_i_ is the isotopic ratio of that fish, *δ* ^15^N_base_ is the isotopic ratio of the primary consumers (macro‐invertebrates), 3.4 is the fractionation between trophic levels and 2 is the trophic position of the baseline organism (Post 2002).

The stable isotope data were initially used in linear mixed models to assess differences between the species, and their allopatric and sympatric treatments. Species were entered into models according to their treatments so, for example, *B. barbus* was present in models as (1) allopatric *B. barbus*; and (2) in sympatry with *S. cephalus*. The dependent (response) variable was *δ* ^13^C or *δ* ^15^N and each model was fitted with mesocosm number as a random effect on the intercept. This was to prevent inflation of the residual degrees of freedom that would occur had each individual fish been used as a true replicate (Tran et al. 2015). The differences in the stable isotope values by species and treatment were determined using estimated marginal means and linearly independent pairwise comparisons with Bonferroni correction for multiple comparisons. A similar linear mixed model approach was also used to test for differences in the initial fish lengths between the species and their treatments, and to assess differences in IL between treatments per species at the end of the experiment, with the same model structure used.

The stable isotope data were then used to calculate the trophic niche sizes of both species per treatment using the metric ‘standard ellipse area’ (SEA_c_; the subscript ‘c’ indicates a small sample size correction). These calculations were completed in the SIAR package (Jackson et al. 2011) in the R computing program (R Development Core Team 2011). The data from each mesocosm were combined for each treatment, as there were no differences between their isotopic baselines due to the enclosures being placed in the same pond. SEA_c_ is a bivariate measure of the distribution of individuals in their trophic space, with the models used enclosing 60% of the data. Thus, SEA_c_ represented the core dietary niche of that population (hereafter referred to as the trophic niche) (Jackson et al. 2011, 2012). Where SEA_c_ overlapped between the sympatric fishes within a treatment, then the area and percentage of *B. barbus* overlap with *S. cephalus* was also calculated to indicate the extent of actual resource sharing. In addition, this overlap was also calculated for each combination of species in their allopatric contexts in order to demonstrate their potential niche overlap and enable comparison with their realized niche overlap in sympatry. These comparisons were possible due to the similarity of the habitats and prey items within the enclosures, the result of their placement within one larger pond.

### Streams

**Figure 1:**
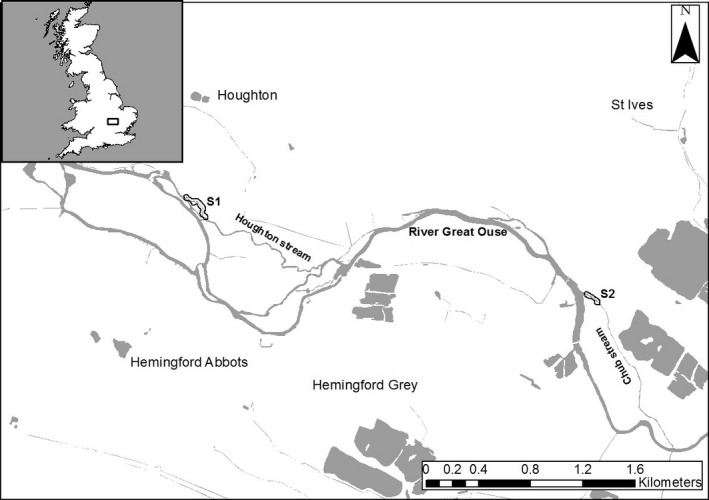
Location of the streams used in the *Barbus barbus* stocking field experiment. Inset: Approximate locations of the streams in Britain. Main map: location of the Houghton stream and Chun stream in relation to the main River Great Ouse and where S1 and S2 represent the stocking locations (OS Open Map – Local 2015).

Assessment of the trophic consequences of stocking hatchery‐reared *B. barbus* for resident *S. cephalus* and other fishes was completed in two streams connected to the River Great Ouse. These were the Houghton Stream (52.328607, −0.116417; Fig. 1) and the St. Ives Chub Stream (hereafter referred to as the Chub Stream; 52.321542, −0.072521; Fig. 1). The source of both streams was an outflowing connection from the main River Great Ouse. They both then flowed for approximately 1500 m before rejoining the main river. Both streams were 6–10 m in width with depths to 2 m, and comprised of pool and riffle habitat. The Great Ouse at either end of the streams was canalized with highly regulated flows.

Given the low probability of recapturing marked fish in these wild situations, growth assessments were not included in this aspect of the study. Thus, the focus was only on assessing the trophic interactions between the resident fishes and stocked *B. barbus*. While *B. barbus* is indigenous to the Great Ouse catchment (Antognazza et al. 2016), the two streams were located at least 30 km downstream of the reaches where *B. barbus* populations were prevalent. However, their flow regimes, habitats and substrates were all considered suitable for *B. barbus* and so fishery managers were trying to establish populations in these streams that had a resident fish community dominated in biomass by *S. cephalus*. Approximately, 500 hatchery‐reared *B. barbus* (100–150 mm; age 1+) were released in December 2013 into each stream. A subsequent release of 1000 fish was also completed in December 2014. The recapture of these fishes was completed using electric fishing, completed in July to August 2014 and June to September 2015. Due to the habitat of the streams, a combination of wading and electric fishing from a boat was used, with hand‐held equipment used throughout. With the focus being in recapturing stocked fish for stable isotope analysis, fishing was qualitative and so did not utilize stop‐nets or incorporate population estimates. All the major stream habitats were sampled. All captured fish were identified to species, measured (fork length, nearest mm) and between 3 and 5 scales removed. They were then released back into the streams. Concomitantly, macro‐invertebrate samples were collected using kick sampling.

The trophic relationships of the fishes from each sampling occasion were assessed using stable isotope analysis. There were two differences from the methods used for the mesocosm experiment. First, for the fishes, stable isotope data were derived from scales rather than dorsal muscles (Busst et al. 2015; Busst and Britton 2016). As it is only the outer proportion of scales that reflect the recent growth of the fish and thus their recent isotopic values, then in all cases only the very outer edge of the scales were removed and analyzed (Grey et al. 2009). Second, to account for differences in the isotopic baseline between years in the streams, the stable isotope data were corrected for these isotopic differences. This process removes the annual variability in the consumer isotope data caused by the annual variation in their putative food sources, so enabling accurate comparison in their metrics (Olsson et al. 2009). The *δ* ^15^N data were transformed to trophic position (TP) as previously described, while *δ* ^13^C was corrected according to: *δ* ^13^Ccorr = *δ* ^13 ^C_i_ – *δ* ^13^C_meaninv_/CR_inv_, where *δ* ^13^C_corr_ is the corrected carbon isotope ratio of the individual fish, *δ* ^13 ^C_i_ is the uncorrected isotope ratio of that fish, *δ* ^13^C_meaninv_ is the mean invertebrate isotope ratio (the ‘baseline’ invertebrates) and CR_inv_ is the invertebrate carbon range (*δ* ^13^C_max_ – *δ* ^13^C_min_) (Olsson et al. 2009). Standard ellipse area (SEA_c_) for each species and the extent of *B. barbus* overlap with resident fishes were then calculated as per the mesocosm experiment. Wherever possible, only fishes of similar lengths were compared for their trophic niche sizes and overlap to prevent confounds relating to ontogenetic shifts in fish diet.

### Lowland rivers

The trophic niche breadths and overlaps of *B. barbus* and *S. cephalus* were then assessed in lowland rivers to determine whether the patterns observed at smaller spatial scales were apparent in more complex situations. Three rivers were used, two sections of the River Great Ouse, the River Lea and River Avon. The Lea and Great Ouse have indigenous *B. barbus* populations while the Avon population is nonindigenous but established for over 100 years (Antognazza et al. 2016). All the rivers have received stockings of hatchery‐reared *B. barbus* in the last 20 years, although it could not be determined whether the fish analyzed here were of wild or hatchery origin.

**Figure 2:**
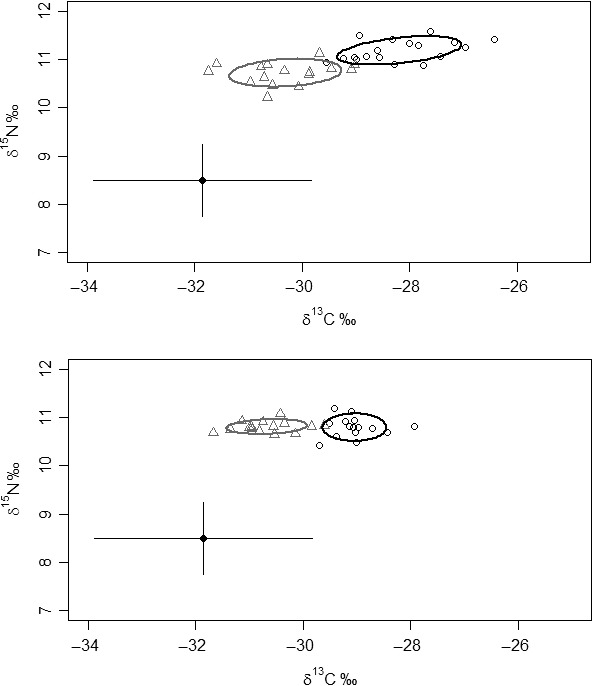
Stable isotope bi‐plots for the mesocosm experiment, where (○) *Barbus barbus* individuals, (Δ) *Squalius cephalus* individuals and (●) mean (± SE) values of putative macro‐invertebrate food resources. Solid lines enclose the standard ellipse areas for each species, where black: *B. barbus*, dark grey: *S. cephalus*. Top: species in allopatry; Bottom: species in sympatry.

**Figure 3:**
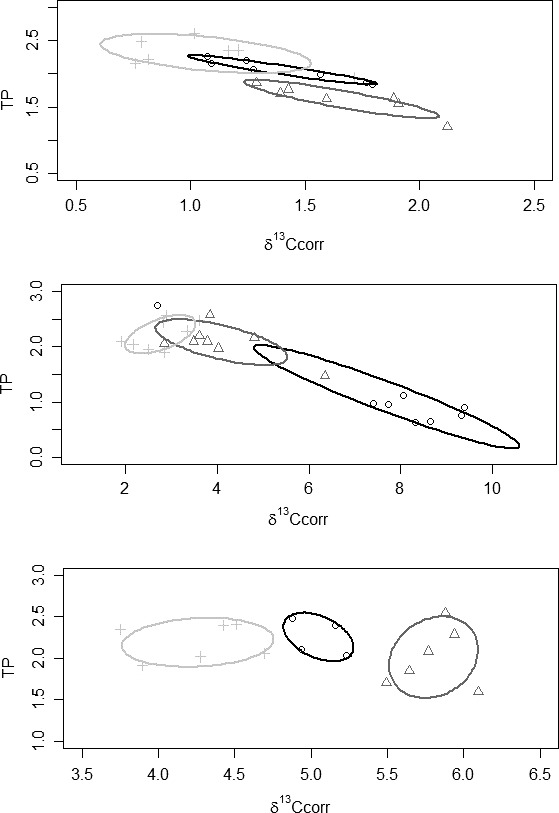
Stable isotope bi‐plots for the Chub stream where (○) *Barbus barbus* individuals, (Δ) *Squalius cephalus* individuals and (+) *Leuciscus leuciscus* individuals. Solid lines enclose the standard ellipse areas for each species, where black: *B. barbus*, dark grey: *S. cephalus*, light grey: *L. leuciscus*. Note the different scales on the axes. Top: June/August 2014; Middle: June 2015; Bottom: September 2015.

**Figure 4:**
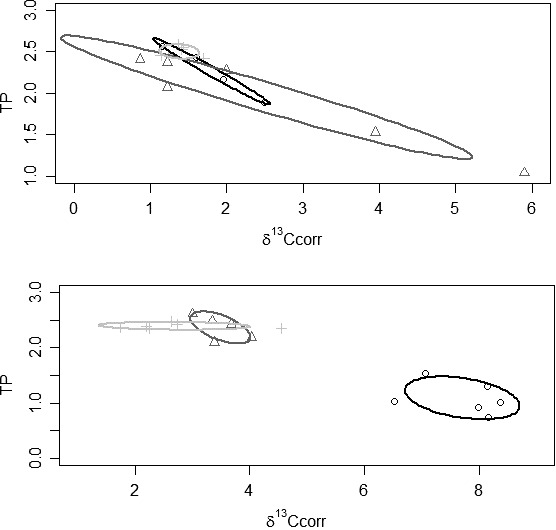
Stable isotope bi‐plots for the Houghton stream where (○) *Barbus barbus* individuals, (Δ) *Squalius cephalus* individuals and (+) *Leuciscus leuciscus* individuals. Solid lines enclose the standard ellipse areas for each species, where black: *B. barbus*, dark grey: *S. cephalus*, light grey: *L. leuciscus*. Note the different scales on the axes. Top: June/August 2014; Bottom: June 2015.

The two sites on the Great Ouse were at Newport Pagnell (Site 1: 52.088232, −0.714125; Fig. 2) and Odell (Site 2: 52.209929, −0.584748; Fig. 2). These sites were both approximately 100 m in length and up to 20 m wide, and comprised of large pool‐riffle habitat. The site on the River Lea was at Batford (51.821735, −0.337205; Fig. 3). The sampled site was approximately 100 m in length, with widths up to 12 m. The habitat comprised of smooth flowing glides. Both rivers were sampled by electric fishing from a boat in July 2014. Due to their size, qualitative approaches were used with no stop‐nets. The data collected were as described for the side channels, although an invertebrate sample was unable to be collected from the River Lea. For the River Avon, fish samples were collected by angling from Ellingham (50.874070, −1.804103; Fig. 4), with an invertebrate baseline collected by kick sampling. In all cases, the sizes of the fishes sampled from these sites were considerably larger than those used experimentally and in the side channels. At all sites, fish lengths were recorded (fork length, nearest mm) and scale samples taken. These scales were then used in the stable isotope analysis, using the methodology already outlined for the streams. The stable isotope metrics of trophic niche size (as SEA_c_) and trophic overlap were then compared between the *B. barbus* and *S. cephalus* within each site. This meant there was no requirement to correct the data and so all the stable isotope analyses were completed as per the mesocosm experiment.

## Results

### Pond mesocosm experiment

There were no significant differences in the starting length ranges of the fish (LMEM, *P *=* *0.09; Table 1). At the conclusion of the experiment, 95% of the fish that were introduced into the enclosures were recovered. The maximum number of fish missing from a mesocosm was one and it was assumed that these individuals had died during the experiment. The LMEM testing for differences in the final lengths of these fishes revealed that the overall model was significant (*P *<* *0.01). The pairwise comparisons indicated that the significant differences were only between *B. barbus* and *S. cephalus*, irrespective of the treatment (*P *=* *0.02 in allopatry and *P *<* *0.01 in sympatry). There were no significant differences in the final lengths of each species between their allopatric and sympatric contexts (*P *>* *0.10; Table 1). When converted to IL, the 95% confidence range for *B. barbus* in allopatry was 0.98–1.10 mm day^−1^ and in sympatry 0.98–1.09 mm day^−1^. For *S. cephalus*, this was 1.01–1.17 mm day^−1^ in allopatry and 1.02–1.17 mm day^−1^ in sympatry. Thus, there were no significant differences in growth rate between the treatments in each species.

**Table 1:** Number of fishes analyzed, the mean starting fork lengths, the mean incremental lengths (IL), mean *δ* ^13^C, mean *δ* ^15^N, trophic position (TP), and trophic niche size (as standard ellipse area corrected for small sample size, SEA_c_) of *B. barbus* and *Squalius cephalus* at the conclusion of the mesocosm experiment and the extent to which *B. barbus* trophic niche overlapped (%) with *S. cephalus*. Error around the mean represents standard error

Species	Treatment	*n*	Mean starting length (mm)	Mean IL (mm day^−1^)	Mean *δ* ^13^C (‰)	Mean *δ* ^15^N (‰)	Mean TP	SEA_C_ (‰^2^)	Overlap (%)
*B. barbus*	Allopatry	18	77.6 ± 0.96	0.34 ± 0.03	−28.2 ± 0.20	11.2 ± 0.05	2.79 ± 0.02	0.56	
	Sympatry	15	77.5 ± 1.31	0.41 ± 0.03	−29.1 ± 0.11	10.8 ± 0.05	2.68 ± 0.02	0.31	0
*S. cephalus*	Allopatry	17	73.9 ± 1.22	0.45 ± 0.05	−30.3 ± 0.19	10.7 ± 0.05	2.66 ± 0.02	0.54	
	Sympatry	15	76.1 ± 1.60	0.50 ± 0.01	−30.7 ± 0.14	10.8 ± 0.03	2.68 ± 0.01	0.21	0

The influence of species and treatment on the stable isotope data was significant for both *δ* ^13^C and *δ* ^15^N (*P *<* *0.01 in all cases; Table 2). For *δ* ^13^C, significant differences between the species were evident between their allopatric contexts and when they were in sympatry (*P *<* *0.01, Tables 1, 3); *S. cephalus* was depleted in *δ* ^13^C compared to *B. barbus*. For *δ* ^15^N, when analyzed as trophic position, there was a significant difference between the species in allopatry (*P* < 0.01). There was no significant difference in TP between the species in sympatry (*P* > 0.10; Tables 1, 3). Regarding SEAc, both species had larger trophic niches in allopatry than in sympatry, with no overlap between them in both contexts (Table 1; Fig. 2). Additionally, *B. barbus* had a considerably larger trophic niche than *S. cephalus* in both allopatry and sympatry (Table 1).

**Table 2:** Outputs and significance of the final linear mixed models testing the differences in mean *δ* ^13^C and trophic position (TP) between the species across the mesocosm experiment, where mesocosm was the random effect on the intercept

Final model structure (and result):			
*δ* ^13^C ~ species × experimental treatment (AIC = 141.8; log likelihood = −64.9; *P *<* *0.01)			
Trophic position ~ species × experimental treatment (AIC = −178.9; log likelihood = 95.4; *P *<* *0.01)			
Pairwise comparison		Mean difference in *δ* ^13^C	Mean difference in TP
Allopatric	Allopatric *Squalius cephalus*	2.12 ± 0.36, *P *<* *0.01*	0.13 ± 0.03, *P *<* *0.01*
*B. barbus*	Sympatric with *S. cephalus*	0.85 ± 0.36, *P *>* *0.1	0.11 ± 0.03, *P *=* *0.01*
Allopatric	Sympatric with *B. barbus*	0.36 ± 0.36, *P *>* *0.1	0.02 ± 0.03, *P *>* *0.1
*S. cephalus*			
*B. barbus* in sympatry with *S. cephalus*		1.63 ± 0.23, *P *<* *0.01*	0.004 ± 0.02, *P *>* *0.1

Mean differences are from estimated marginal means (* = difference significant at *P *<* *0.05).

**Table 3:** Date of sampling, species, sample sizes, mean fork lengths, mean *δ* ^13^C and mean *δ* ^15^N of fish and their trophic niche size (SEAc*; values obtained from data corrected for baseline variations across treatments.) and the extent to which *B. barbus* trophic niche overlaps (%) with other fish species in the community (*Squalius cephalus* and *Leuciscus leuciscus*), at (A) Chub stream and (B) Houghton stream. Error around the mean is standard error

Date	Species	*n*	Mean length (mm)	Mean *δ* ^13^C (‰)	Mean *δ* ^15^N (‰)	SEAc (‰^2^)*	Overlap (%)
(A)							
June 2014	*B. barbus*	7	209.9 ± 9.9	−27.1 ± 0.3	16.2 ± 0.2	0.06	
	*S. cephalus*	7	217.4 ± 5.7	−26.4 ± 0.3	14.7 ± 0.3	0.11	<0.01
	*L. leuciscus*	7	203.1 ± 2.6	−28.1 ± 0.4	17.0 ± 0.3	0.24	0.40
June 2015	*B. barbus*	8	151.1 ± 6.5	−22.3 ± 0.9	13.3 ± 0.8	1.66	
	*S. cephalus*	8	153.6 ± 8.0	−26.4 ± 0.4	16.6 ± 0.4	0.90	0
	*L. leuciscus*	8	152.6 ± 9.6	−27.9 ± 0.2	17.1 ± 0.3	0.44	0
Sept 2015	*B. barbus*	4	212.0 ± 20.9	−27.5 ± 0.1	18.6 ± 0.4	0.16	
	*S. cephalus*	6	209.2 ± 15.3	−26.9 ± 0.1	17.8 ± 0.5	0.30	0
	*L. leuciscus*	6	184.8 ± 6.6	−28.2 ± 0.1	18.4 ± 0.3	0.31	0
(B)							
June 2014	*B. barbus*	4	185.3 ± 9.2	−28.2 ± 0.4	17.1 ± 0.5	0.12	
	*S. cephalus*	6	194.8 ± 6.2	−27.3 ± 1.0	16.0 ± 0.8	1.07	0.58
	*L. leuciscus*	6	191.7 ± 3.9	−28.7 ± 0.1	17.9 ± 0.1	0.05	0.17
June 2015	*B. barbus*	6	159.0 ± 8.8	−22.8 ± 0.3	13.4 ± 0.4	0.77	
	*S. cephalus*	5	198.4 ± 23.7	−27.5 ± 0.2	17.7 ± 0.3	0.28	0
	*L. leuciscus*	6	161.7 ± 15.1	−28.4 ± 0.5	17.8 ± 0.1	0.20	0

### Streams

Across the surveys of the two streams, three fish species were studied, *B. barbus*,* S. cephalus* and dace *Leuciscus leuciscus* (Table 3). While the fish were considerably larger than used in the mesocosm experiments, mean lengths per species were all between 151 and 217 mm (Table 3). Sample sizes tended to be small, especially for *B. barbus*, where only 10 stocked fish were captured in subsequent sampling in the Houghton Stream and 19 in the Chub Stream (Table 3). Although there was some temporal variability in the stable isotope data in each stream, there was a general pattern of minimal trophic overlap between stocked *B. barbus* and the resident *S. cephalus* and *L. leuciscus* (<1%) with this particularly apparent in samples collected in 2015 (Table 3; Figs. 3, 4).

### Lowland rivers

**Figure 5:**
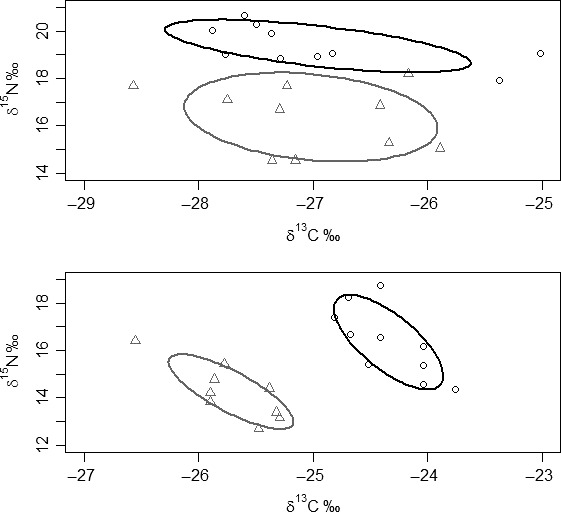
Stable isotope bi‐plots for the River Lea where (○) *Barbus barbus* individuals, (Δ) *Squalius cephalus* individuals. Solid lines enclose the standard ellipse areas for each species, where black: *B. barbus*, dark grey: *S. cephalus*. Note differences in scales on all axes. Top all fish between 186 and 237 mm; Bottom: all fish between 400 and 435 mm.

**Figure 6:**
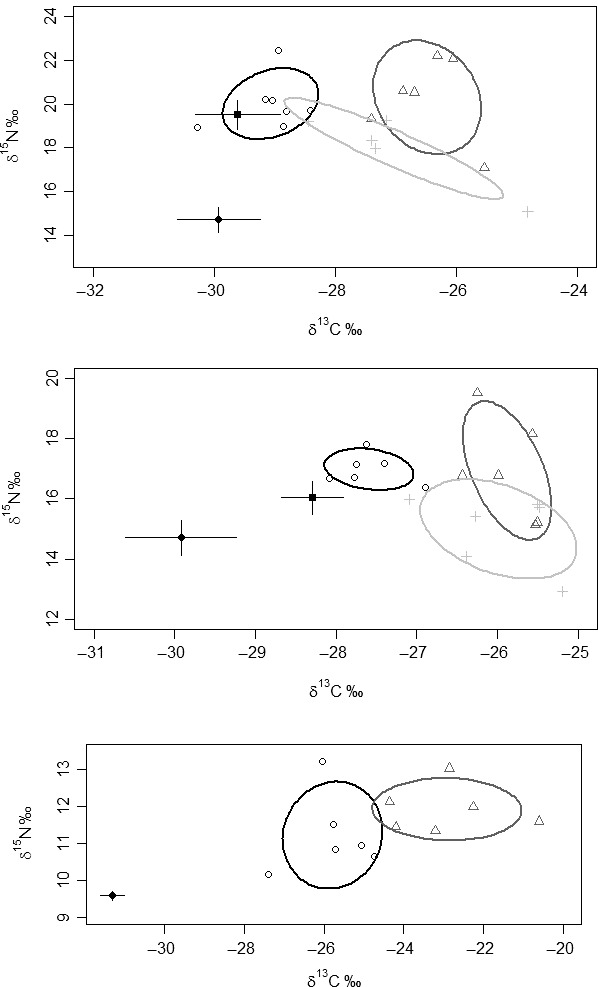
Stable isotope bi‐plots for the Site 1 (Top) and 2 (Middle) on the Great Ouse, and the River Avon (Bottom), where (○) *Barbus barbus* individuals, (Δ) *Squalius cephalus* individuals and (+) *Leuciscus leuciscus* individuals with mean (± SE) values of putative food sources: macro‐invertebrates (●) and signal crayfish (■). Solid lines enclose the standard ellipse areas for each species, where black: *B. barbus*, dark grey: *S. cephalus*, light grey: *L. leuciscus*. Note the different scales on the axes.

The fish sampled across the three rivers tended to be the largest used in the study, with some *B. barbus* present in samples >600 mm (Table 4). In the River Lea, two size classes of *B. barbus* and *S. cephalus* were present and so were analyzed and tested separately. As with the second pond mesocosm experiment and the side channels, the extent of the trophic overlap of *B. barbus* with other cyprinid species was minimal (Table 4; Figs. 5, 6). This was the case for both size classes of fish in the River Lea, although there was some shift in this pattern between the size classes (Fig. 5). In the fish of lengths 186–237, the *B. barbus* stable isotopes were nitrogen enriched by approximately 3‰ compared to *S. cephalus*, but had similar values of *δ* ^13^C (Table 4; Fig. 5). By contrast, for the fish of above 400 mm, the *B. barbus* has enriched *δ* ^13^C and *δ* ^15^N compared to *S. cephalus* (Table 4; Fig. 5).

**Table 4:** Species, sample sizes, mean fork lengths, mean *δ* ^13^C and mean *δ* ^15^N of sampled fish, their trophic niche breadth (SEA_c_) and the extent to which *B. barbus* trophic niche overlaps (%) with other sampled fishes (*Squalius cephalus* and *L. leuciscus*). Error around the mean is standard error

Site	Species	*n*	Mean length (mm)	Mean *δ* ^13^C (‰)	Mean *δ* ^15^N (‰)	SEAc (‰^2^)	Overlap (%)
Site 1, Great Ouse	*B. barbus*	7	162.6 ± 44.9	−29.1 ± 0.2	20 ± 0.5	2.54	
	*S. cephalus*	6	290.2 ± 70.4	−26.5 ± 0.3	20.3 ± 0.8	4.85	0
	*L. leuciscus*	5	138.4 ± 19.8	−27.0 ± 0.6	18.0 ± 0.8	3.60	<0.01
Site 2, Great Ouse	*B. barbus*	6	252.5 ± 8.4	−27.6 ± 0.2	17.0 ± 0.2	0.79	
	*S. cephalus*	6	346.0 ± 39.6	−25.6 ± 0.2	16.9 ± 0.7	2.32	0
	*L. leuciscus*	6	167.7 ± 1.9	−26.0 ± 0.3	15.0 ± 0.5	3.16	0
Lea (>400 mm)	*B. barbus*	10	415.1 ± 3.9	−24.3 ± 0.1	16.3 ± 0.5	2.21	
	*S. cephalus*	9	415.3 ± 3.8	−25.7 ± 0.1	14.2 ± 0.4	3.87	<0.01
Lea (<250 mm)	*B. barbus*	10	225.5 ± 4.6	−27.0 ± 0.3	19.4 ± 0.3	1.29	
	*S. cephalus*	10	213.9 ± 4.2	−27.0 ± 0.3	16.4 ± 0.4	1.02	0
Avon	*B. barbus*	6	586.7 ± 13.8	−25.8 ± 0.4	11.2 ± 0.4	3.87	
	*S. cephalus*	6	531.7 ± 7.0	−22.9 ± 0.6	11.9 ± 0.3	3.38	0

## Discussion

Experimental and field evidence suggested that there was substantial partitioning in the trophic niches of sympatric *B. barbus* and *S. cephalus*, with no evidence for resource sharing. This pattern was apparent over a 100 days period in the mesocosm enclosures, with this an important result as it was from an experiment completed in relatively controlled conditions. In the field studies, where there is greater inherent complexity and stochasticity in the systems that result in more difficulty in deciphering ecological patterns and thus where more caution is needed in interpretation, the trophic niche partitioning was also apparent. This was the case in the two‐year post‐stocking period in the two streams and in the larger fishes sampled in the lowland rivers. Moreover, where there were data available for other fishes in the community, such as *L. leuciscus*, this pattern of *B. barbus* having a very discrete trophic niche was still evident.

The outputs of the allopatric treatment in the mesocosm experiment suggested that *B. barbus* rapidly established a trophic niche that was divergent from allopatric *S. cephalus*, suggesting that there would be no sharing of food resources when the species were in sympatry. When the species were in sympatry, their actual trophic niches did remain separated. However, their niche breadths were reduced in sympatry, indicating some individual specialization (Araújo et al. 2011). This result was consistent with both the prediction and the niche variation hypothesis that predicts populations become less generalized in more competitive environments (Van Valen 1965; Human and Gordon 1996; Olsson et al. 2009). Similar patterns of trophic niche divergence and partitioning have been detected when non‐native fishes that have been introduced into similar environments. For example, the trophic niche divergence between the small, invasive fish topmouth gudgeon *Pseudorasbora parva* with extant species, including carp *Cyprinus carpio*, facilitates their coexistence (Jackson and Britton 2013; Tran et al. 2015). These trophic niche outputs were also important in the context of the growth rates of the fishes. In the mesocosm experiment, the growth rates of both fishes were similar between their allopatric and sympatric treatments, despite their reduced trophic niche sizes. This suggests that when the fishes have access to food resources that are not limiting, their trophic niche partitioning and specializations maintain their energetic requirements to enable their growth rates to be similar between the allopatric and sympatric treatments. This was contrary to the prediction that increased trophic specialization would result in decreased growth rates. This was also an important result given the difficulty of measuring differences in growth rates in more wild situations, such as the field sites, where there tends to be a wide range of abiotic factors that cause temporal and individual variability in fish growth rates (Beardsley and Britton 2012; Liu et al. 2015).

Introduced and stocked salmonid fishes often cause detrimental impacts for native salmonids. Predation by introduced lake trout (*Salvelinus namaycush*) can limit the distribution of bull trout (*Salvelinus confluentus*) (Donald and Alger 1993) and cause population declines of cutthroat trout (*Onchorhynchus clarki*) (Ruzycki et al. 2003). Their stocking can cause trophic cascades (Tronstad et al. 2010) that influence predator–prey interactions in surrounding terrestrial ecosystems (Middleton et al. 2013). For *B. barbus*, however, there was minimal evidence to suggest that their ecological interactions resulted in any substantial alteration in the trophic ecology of *S. cephalus*. It is acknowledged that the approach used within this study was relatively simple, focusing primarily on the trophic interactions of *B. barbus* with *S. cephalus*. This was to ensure that the interspecific comparisons were being made for functionally similar fishes that grew to relatively similar body sizes and that live for similar long life spans (Britton 2007). This could, however, have resulted in some over‐simplification of the outcomes of their stocking into more complex fish communities. However, there is also no evidence of *B. barbus* sharing a trophic niche space with fishes such as *L. leuciscus*, roach *Rutilus rutilus* and graying *Thymallus thymallus*, both here and from other studies (e.g., Bašić and Britton 2015).

The design of the experimental and field studies meant that regular assessment of the trophic niches of the fishes in each system was not possible. Logistical constraints limited the number of treatments that could be included within the mesocosm experiment. This meant that fish numbers, that is, density, was maintained across the experimental treatments. This was important to ensure that comparisons could be made in trophic niche sizes between species and the allopatric and sympatric contexts, as the numbers of fish involved were consistent. However, the partitioning of trophic niches between species can be related to competition for food resources and predation (Nilsson 1967) and thus patterns can change as the population abundances of the species increase (Spurgeon et al. 2015). Although our patterns of partitioning were strong in the mesocosms and were detected in the field studies, it is acknowledged that the incorporation of more complexity into the experimental designs, such as including treatments that increased fish abundance or also used fish of contrasting body sizes, might have provided greater insights. Moreover, the focus here was on the trophic relationships of the fishes, yet the impacts of stocked and invasive fishes can include other ecological issues, including habitat disturbances (Gozlan et al. 2010). Indeed, *B. barbus* act as ‘zoogeomorphic agents’ in rivers, as their foraging activities reduce bed material stability, increase bedload transport, and impact micro‐topographic roughness and sediment structure (Pledger et al. 2014, 2016). Thus, their release into rivers where populations are not currently present could have considerable effects on the substrate. By extension, their foraging activities could also impact aspects of the macro‐invertebrate communities, although again this was unable to be tested here. In addition, while stable isotope data can provide a powerful tool to determine trophic interactions, they are only a proxy for this. Studies that compare the diet of fishes across methods such as stable isotope analysis and stomach contents analysis often show some differences in their results (e.g., Hamidan et al. 2015). Consequently, studies that rely solely on stable isotope analysis should be evaluated with some caution (Locke et al. 2013).

The design of fish stocking strategies needs to consider the survival and establishment of the fishes, and their ecological and genetic interactions with extant populations. Knowledge on these aspects and interactions has been well documented for stocked salmonid fishes (e.g., Simon and Townsend 2003). For fishes from other families, however, there remain considerable knowledge gaps, especially in European lowland rivers. Here, our results suggested that *B. barbus* occupied a trophic niche that was distinct from the other cyprinid fishes analyzed. Although this has the caveat around the limitations of the study as outlined above, these results suggest that *B. barbus* stocking can result in relatively minor ecological consequences. This is important, as their stocking can provide considerable recreational and socioeconomic benefits (Britton and Pegg 2011). Notwithstanding, Antognazza et al. (2016) did reveal that, genetically, the stocking of *B. barbus* between different river basins does impact their genetic integrity. In combination, this suggests that in designing fisheries management strategies for lowland rivers where communities are dominated by cyprinid fishes, a wide range of abiotic, ecological and genetic issues need to be considered. There should be identification of the current constraints on the fish community (Cowx 1994), and whether habitat restoration and rehabilitation are more appropriate management tools than stocking (Pretty et al. 2003). Should stocking be demonstrated to be a viable management option, then our work on *B. barbus* indicates that both ecological and genetic considerations must be applied to the decision of why, when and how to stock the fishes.

## Conflict of Interest

None declared.
